# Supplementary figures and images for: Metal coordination and enzymatic reaction of the glioma-target R132H isocitrate dehydrogenase 1: Insights by molecular simulations
Source: PLoS One. 2025 Jun 26;20(6):e0326425. doi: 10.1371/journal.pone.0326425 (PMC12200840; doi:10.1371/journal.pone.0326425)

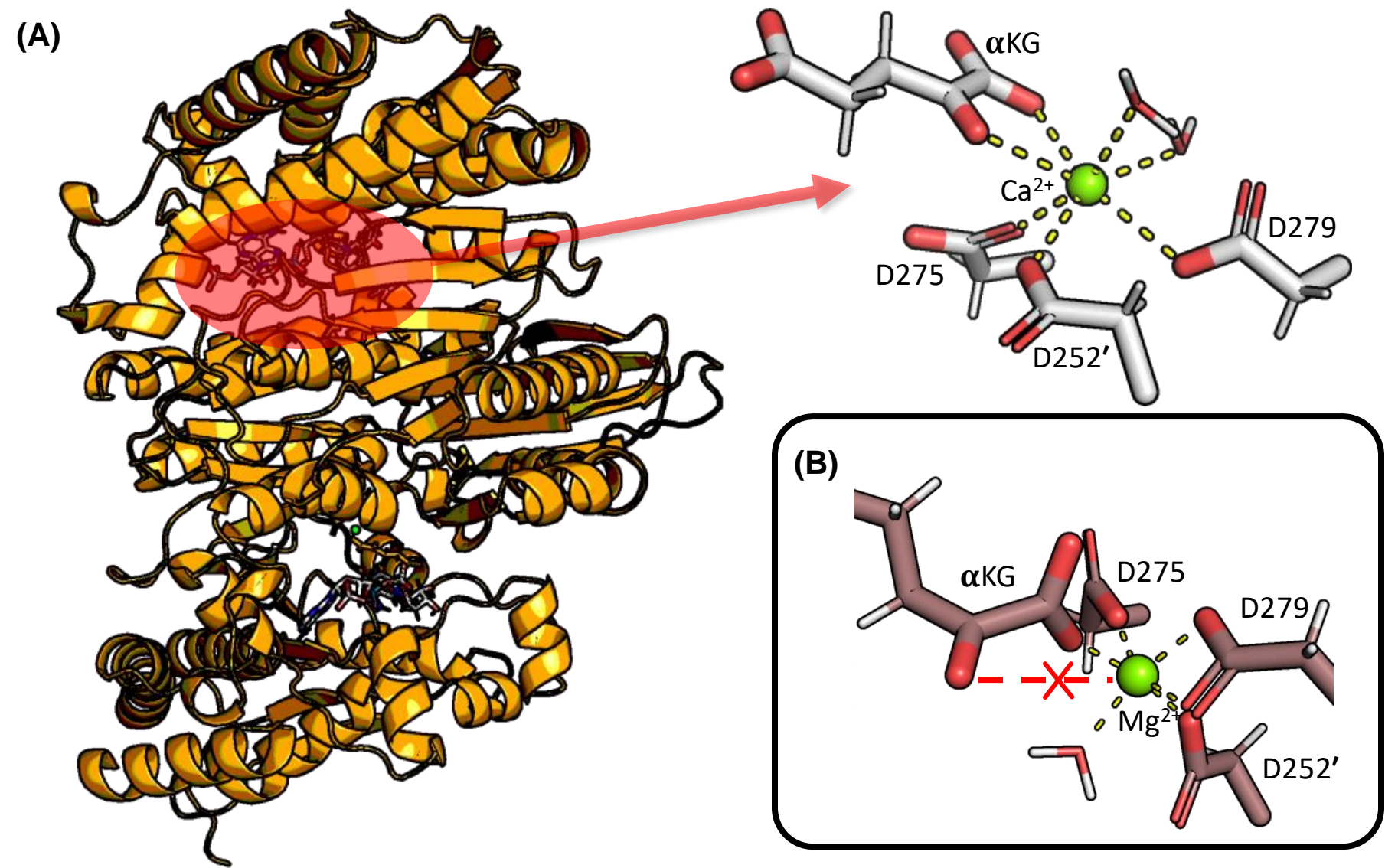

Supplement: S1 Fig [file pone.0326425.s001.pdf]

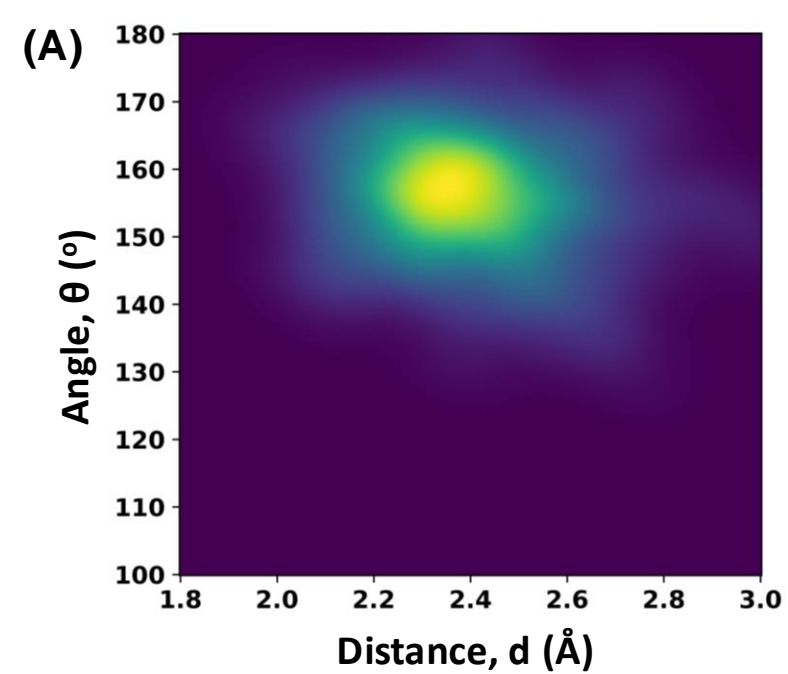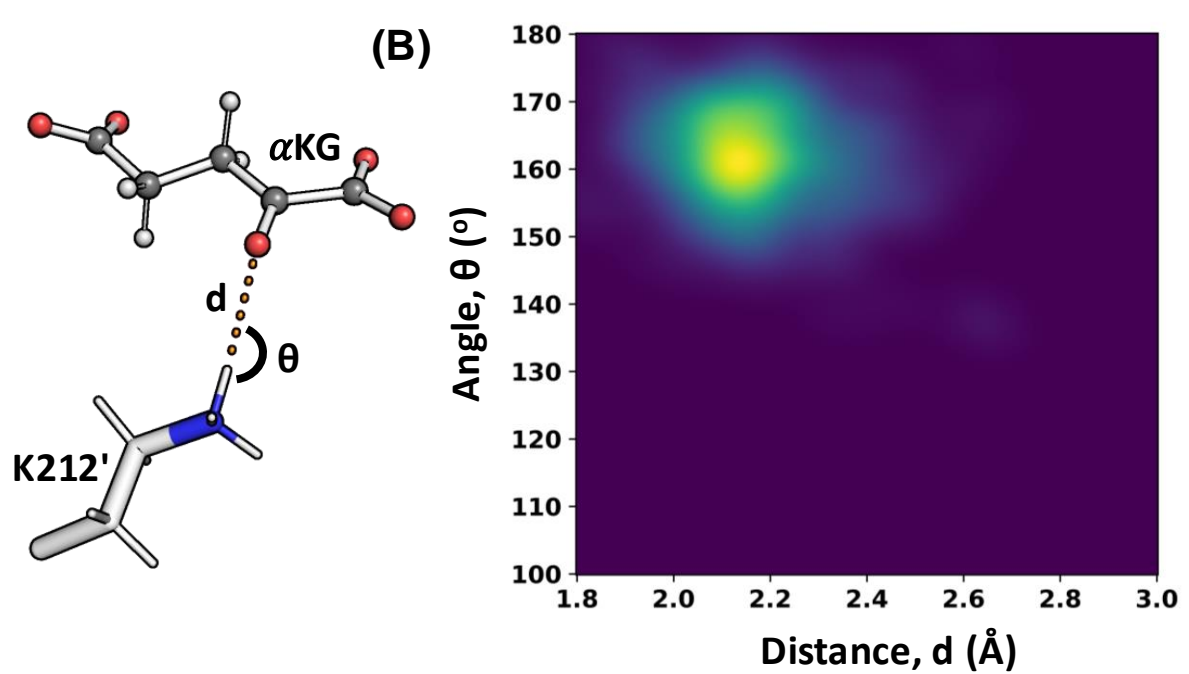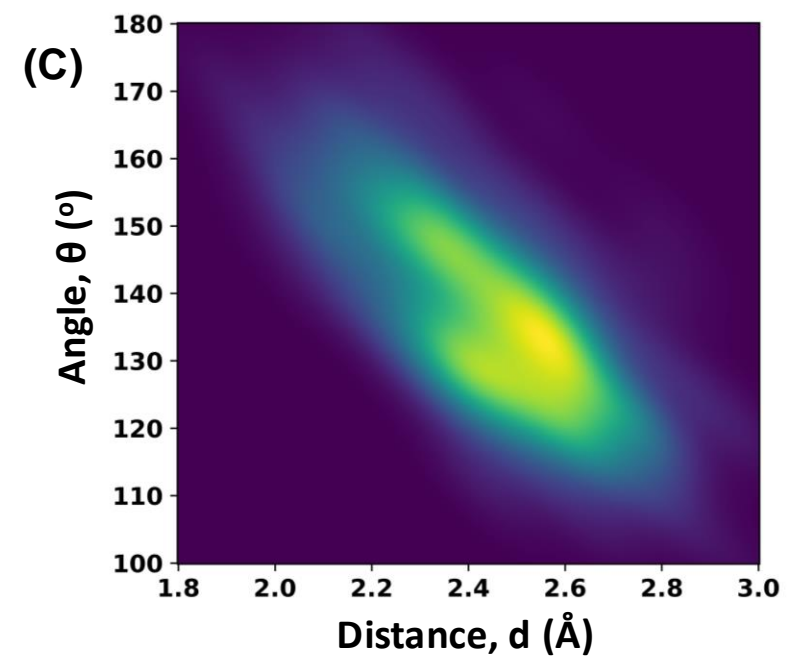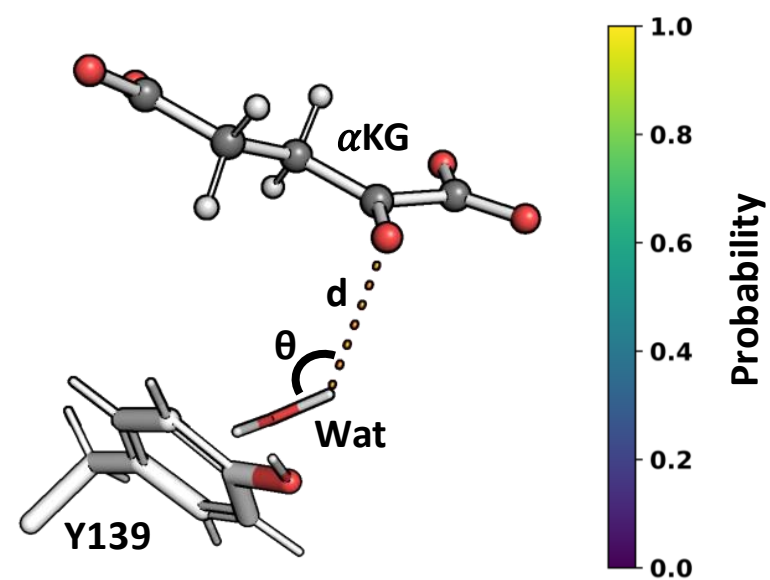

Supplement: S2 Fig [file pone.0326425.s002.pdf]
