# Supplementary material for: Metal coordination and enzymatic reaction of the glioma-target R132H isocitrate dehydrogenase 1: Insights by molecular simulations
Source: PLoS One. 2025 Jun 26;20(6):e0326425. doi: 10.1371/journal.pone.0326425 (PMC12200840; doi:10.1371/journal.pone.0326425)
Supplement: S3 File [file pone.0326425.s003.pdf]

Supplementary Text

Metal Coordination and Enzymatic Reaction of the Glioma-Target R132H Isocitrate Dehydrogenase 1: Insights by Molecular Simulations

Bharath Raghavan[1,2,#a], Marco De Vivo[3], Paolo Carloni[1,4*]

[1] Computational Biomedicine, Institute for Neuroscience and Medicine 9, Forschungszentrum Jülich GmbH, Jülich, Germany
[2] Department of Physics, Rheinisch-Westfälische Technische Hochschule Aachen University, Aachen, Germany
[3] Molecular Modelling and Drug Discovery, Italian Institute of Technology, Genova, Italy
[4] Department of Physics and Universitätsklinikum, Rheinisch-Westfälische Technische Hochschule Aachen University, Aachen, Germany
[#a]Current Address: National Center for Computational Sciences, Oak Ridge National Laboratory, Oak Ridge, United States of America

* Corresponding author Email: p.carloni@fz-juelich.de (PC)

1 Unconstrained Classical MD Simulations of Mut-IDH1

As shown in S1 Fig., 10 ns force-field based molecular dynamics (MD) simulations performed here could not accurately capture the bidentate binding of αKG to the Mg^{2+} ion. This bidentate binding is required for the catalysis, and is expected to be present at the Michaelis complex. [1] The following force field parameters were tested:

1. The standard Mg^{2+} parameters in the Amber99sb*-ildn force field. [2,3] This was also used in our previous work on wt-IDH1. [4]
2. 6-12 Lennard-Jones parameters of Mg^{2+} from Grotz et. al. [5]
3. 6-12 Lennard-Jones parameters of Mg^{2+} from Zhang et. al. [6]

2 QM/MM Regions Used

The final frames from the constrained force-field based MD simulation of 500 ns for both protomers $\mathbf{K/D^H}$ and $\mathbf{K^H/D}$ was used. The QM region used for production QM/MM MD (refered to as **Group II** in the main text) consisted of the cofactor, the substrate, the Mg^{2+} coordination polyhedron, as well as the residues and water involved in αKG binding. The QM regions varied slightly across protomers and acitve sites:

(i) Both active sites of $\mathbf{K^H/D}$ consisted of the residues forming direct or water mediated interactions with αKG, namely: Arg109, Arg100, Lys212', Thr214 (interaction through Wat_1^{rb} and Wat_2^{rb} waters), Thr75 (interaction through 1 water), Ser94, And96. The Mg^{2+} coordination sphere consists of: a water molecule, molecule Asp252, Asp275, a water molecule H-bonded to Asp279. Tyr139, which interacts with Asp275, is also included (185 atoms)

(ii) In active site A of $\mathbf{K/D^H}$, Lys212' does not form an H-bond with αKG. But it is still included in the QM region. Tyr139 H-bonds with a water (refereed to as Wat_{Y139} in the Results section of the main text), that weakly interacts with αKG. This water molecule is also included in the QM region (188 atoms).

(iii) In active site B of $\mathbf{K/D^H}$, Thr75 was rotated away from αKG, and was not included in the QM region. Thr77 forms a water mediated H-bond with αKG. The water molecule was included (179 atoms).

3 Deviation of QM/MM MD from the Crystal Structure

After 20 ps of QM/MM MD for each active site of each protomer, we measured the deviation from the crystal structure. This is shown in Table 1, where the root mean squared deviation of the heavy atoms in the QM region (and excluding waters) from the X-ray structure at the end of the QM/MM MD is shown.

Given the relatively low values, we can summarize that the overall structure of the active site from the X-ray is maintained. This is except for key interaction as described in the main paper.

Protomer	Active Site	RMSD
**K^{H}/D**	A	1.4
	B	1.2
**K/D^{H}**	A	1.2
	B	1.6

Table 1. Root mean square deviation or RMSD (in Å) of the QM region of mut-IDH1 between the last frame of the QM/MM MD simulation and the X-ray structure.

4 Tyr139 and Asp275

In the **K^{H}/D** protomer, Tyr139 is rotated towards Asp275 as in the crystal structure. [1] In the **K/D^{H}** protomer, on the other hand, Tyr139 and Asp275 are rotated away from each other to be in a similar pose to that of wt-IDH1 (Figure 5D in the main text).

Regardless of the protonation state of Lys212', the protons on both Tyr139 and Asp275 are at least 4 Å away from the α-ketone of αKG (Table 2).

Protomer	Active Site	Tyr139	Asp275
**K^{H}/D**	A	4.1±0.3	None
	B	5.5±0.2	None
**K/D^{H}**	A	4.3±0.2	4.0±0.1
	B	4.5±0.2	4.1±0.3

Table 2. Average distance (in Å) from the QM/MM MD simulation of mut-IDH1 between the α-ketonic oxygen of αKG and the proton of Tyr139 and Asp275.

At times, Tyr139 established a water-mediated H-bond interaction with the ketone in active site A of the **K/D^{H}** protomer. This water is refered to as Wat_{Y139} in the main text. Because of its geometry (see the plot of the angle vs distance of αKG and Lys212'/Tyr139 interactions in S2C Fig.), we consider this a very weak H-bond. Ty139-water pair then is not capable of acting as an acid to catalyze the mut-IDH1 reaction.

Supporting Information

S1 Fig. (A) Cartoon representation of the mut-IDH1 with the active site containing the heptacoordinated Ca^{2+} coordination sphere. The αKG substrate is coordinated to Ca^{2+} in a bidendate fashion. (B) Loss of the bidendate coordination of αKG during MD simulations based on the Amber99sb*-ildn force field [2,3].

S2 Fig. The probability distributions of the angle vs distance for Lys212'–α-ketone of αKG interactions (A) in active site A, and (B) in active site B of the **K^{H}/D** protomer, (C) the α-ketone of αKG and Tyr139-water in active site A of the **K/D^{H}** protomer. Because of its geometry, this last interaction cannot be considered as an H-bond. The distributions have been calculated as a kernel-density estimate using Gaussian kernels, starting from 10 ps of the QM/MM MD.

References

1. Dang L, White DW, Gross S, Bennett BD, Bittinger MA, Driggers EM, et al. Cancer-associated IDH1 mutations produce 2-hydroxyglutarate. Nature. 2009;462(7274):739–744.

2. Best RB, Hummer G. Optimized Molecular Dynamics Force Fields Applied to the HelixCoil Transition of Polypeptides. J Phys Chem B. 2009;113:9004–9015. doi:10.1021/jp901540t.

3. Lindorff-Larsen K, Piana S, Palmo K, Maragakis P, Klepeis JL, Dror RO, et al. Improved side-chain torsion potentials for the Amber ff99SB protein force field. Proteins. 2010;78:1950–1958. doi:10.1002/prot.22711.

4. Raghavan B, Paulikat M, Ahmad K, Callea L, Rizzi A, Ippoliti E, et al. Drug Design in the Exascale Era: A Perspective from Massively Parallel QM/MM Simulations. Journal of Chemical Information and Modeling. 2023;63(12):3647–3658.

5. Grotz KK, Cruz-León S, Schwierz N. Optimized Magnesium Force Field Parameters for Biomolecular Simulations with Accurate Solvation, Ion-Binding, and Water-Exchange Properties. Journal of Chemical Theory and Computation. 2021;17(4):2530–2540.

6. Zhang Y, Jiang Y, Peng J, Zhang H. Rational Design of Nonbonded Point Charge Models for Divalent Metal Cations with Lennard-Jones 12-6 Potential. Journal of Chemical Information and Modeling. 2021;61(8):4031–4044.
