# Supplementary material for: Metal coordination and enzymatic reaction of the glioma-target R132H isocitrate dehydrogenase 1: Insights by molecular simulations
Source: PLoS One. 2025 Jun 26;20(6):e0326425. doi: 10.1371/journal.pone.0326425 (PMC12200840; doi:10.1371/journal.pone.0326425)
Supplement: Attachment — Submitted filename: Response to reviewers.pdf [file pone.0326425.s004.pdf]

Reply to the reviewers.

Reviewer #1: Here the Carloni team addresses a highly relevant and acute problem: the R132H mutation in Isocitrate Dehydrogenase 1 (IDH1) is a vital target in multiple brain cancers. IDH1 links metabolism to epigenetics, and as such plays a crucial role in these cancers, causing widespread changes in the epigenetic landscape of these tumors, through histone and DNA demethylases. IDH1/2 mutations can lead to hypermethylated histones and DNA which changes gene expression, commonly by overexpression. Despite the recognition of their critical tumorigenic role, still no drug leads and radiotracers which selectively bind only to the mutant over the wild type are so far lacking.

Here, through comprehensive computational analysis, the authors predicted the structural determinants of the Michaelis complex of this mutant using QM/MM MD. They observe important differences from the X-ray structure, ranging from metal coordination to the positioning of key residues at the active site. Especially, the K212 mutation appears as the likely proton donor in the key proton-transfer step of the R132H IDH1 catalytic reaction. The authors then discover that the same residue in its deprotonated state appears involved in the reaction catalyzed by the wild-type enzyme, albeit via different mechanisms.

The rigor of the work, its implications to drug discovery, and the new protocol that they employ, make me suggest accepting the manuscript as is. This is reenforced by the clarity of the presentation, text and figures.

We thank the reviewer for highly praising our manuscript.

Reviewer #2: This manuscript presents a QM/MM-based molecular simulation study of the active-site architecture and mechanistic features of the R132H mutant of human isocitrate dehydrogenase 1 (IDH1). By comparing their simulated structure to both the wild-type and the inactive crystal structure of the mutant, the authors make a case that the proton donor in the mutant's reaction is most likely Lys212'—a result with potential implications for selective drug design. Overall this is a well-designed and executed study with a number of strengths including the biological and therapeutic relevance and along with the open availability of all the QM/MM input files and trajectories is commendable.

We are grateful for the reviewers' careful consideration of our work and their constructive feedback.

The one minor concern of this reviewer is that the was little exploration of alternative proton donors: While the simulations suggest Lys212' is a key proton donor, it might be helpful for readers if the authors at least mentioned the other potential donors (e.g., Tyr139, Asp275) under different solvent or pH conditions could be critical. While presenting additional free energy or reaction-path calculations could further solidify the argument that Lys 212 is critical, this is not necessary for publication.

We now add the following sentence (in red color) to the last sentence in the discussion:

"Thus, the same residue appears to be well positioned to perform proton transfers in both wt- and mut-IDH1 isoforms (compare the position of Lys212' vis a vis the substrate in Figs 5A and D). This information is not provided by previous quantum chemical studies of the reaction.

[10] However, while the simulations do suggest a key role for Lys212', our calculations do not rule out that other residues (such as, for instance, Tyr139), under different solvent and/or pH conditions, could act as proton donors in the enzymatic reaction"
